# A prospective study on health seeking behaviour and post exposure prophylaxis received by animal bite victims at the anti-rabies clinic in a tertiary care centre of urban Bangalore

**DOI:** 10.12688/f1000research.145673.1

**Published:** 2024-03-11

**Authors:** Jithin Surendran, Ravish HS, Nitu Kumari, Ramya M Prasanth, Nidhi Fotedar

**Affiliations:** 1 Department of Community Medicine, Kasturba Medical College, Mangalore, Manipal Academy of Higher Education, Manipal, Karnataka, India; 2 Department of Community Medicine, Kempegowda Institute of Medical Sciences, Bangalore, Karnataka, 560002, India; 3 Department of Community Medicine, University College of Medical Sciences, New Delhi, 110095, India; 4 Department of Community Medicine, Sri Siddhartha Institute of Medical Sciences & Research Centre, Karnataka, 527107, India

**Keywords:** animal bite victims, compliance, efficacy, health seeking behaviour, post-exposure prophylaxis

## Abstract

**Background:**

Rabies is 100% preventable by administering early and complete post exposure prophylaxis (PEP). Animal bite victims must have the knowledge and attitude necessary to seek appropriate medical care at the earliest to receive the required PEP.

**Objectives:**

The present study sought to ascertain the health-seeking behavior of animal bite victims, their knowledge and attitude regarding rabies prophylaxis, the PEP they received, and their level of compliance with the full course of anti-rabies vaccination.

**Methods:**

The study included animal bite cases that presented to the anti-rabies clinic and matched the eligibility criteria. All the required details were recorded using an internally validated structured questionnaire. All participants were followed up for six months to ensure their health conditions and compliance with the vaccination schedule.

**Results:**

Out of 1058 respondents, 57.9% were adults, with 46.6% belonging to middle socioeconomic class. 91.1% of them were informed biting animals as dogs. Before arriving at the anti-rabies clinic, 93.3% of the study subjects washed their wounds, and 62.4% visited to another health facility. Rabies knowledge was inadequate among the study participants, only 54.8% being mindful about the disease and its prevention. The compliance with the full course of antirabies vaccination was found to be 77.9%. All subjects were healthy, confirming that PEP is safe and effective.

**Conclusion:**

Regular social and behavioral change communication (SBCC) needs to be implemented with regard to health-seeking behavior.

## Introduction

Animal exposure among humans is a public health problem and poses a potential threat of rabies infection to over 3.3 billion people worldwide. ^1^ These exposures occurs mainly in underserved populations, both in rural and urban areas, and has been documented for more than 4000 years. ^2^ The magnitude and epidemiological pattern differ from country to country. According to data available, most cases occur in Africa and Asia, where children represent about 40% of population exposed to dog bites in those rabies endemic areas. ^3^ A combination of large human and dog populations in congested habitable areas has led to more exposure in WHO’s South East Asia region than in any other part of the world. ^4^

In the World Health Organization’s (WHO) South-East Asia (SEA) Region, which includes 11 nations, eight are rabies-endemic, and more than 1.4 billion people are at risk of contracting the disease. As a consequence, it remains a significant public health and economic challenge throughout the region. ^5^ Each year, an estimated 12 million people in Asia seek treatment after being exposed to probable rabies-infected animals. Fear and anxiety following a dog bite result in a psychological consequence that is estimated to account for approximately 1,40,000 DALYs in Asia. ^6^

An estimated 17.4 million animal bites occur in India each year, having a 1.7% incidence. ^7^ In a rabies-endemic nation like India, where every animal bite is suspected of being a rabid exposure, exposed persons should engage in proper health-seeking behavior in order to get early and comprehensive post-exposure prophylaxis (PEP) for rabies antibodies. ^8^ The knowledge and attitude regarding the disease prevention among the bite victims is important for their health seeking behaviour. ^9^

The act of seeking medical assistance is “any action undertaken by individuals who perceive themselves to have a health problem or to be ill for the purpose of finding an appropriate remedy.” It is not an isolated event determined by individuals concentrating solely on their own self-interest; it is “part and parcel of individuals, families, or community’s identity, which involves a combination of social, personal, cultural, and experiential factors”. It is also part of the more general concept of health behavior, which includes behaviors carried out to preserve good health, refrain from illness, and cope with any deviation from a good state of health.

At the health care facility, whenever a patient comes for a health problem such as animal exposure; the post exposure prophylaxis should be provided depending upon the severity of the animal bite wounds/exposures as per the WHO/NCDC, India guidelines. ^10^ The PEP consists of thorough wound washing with soap or detergent & water and application of virucidal agents to reduce the viral inoculum at the wound site; post exposure anti-rabies vaccination to induce antibodies which prevents the risk of virus entering peripheral nerves after a bite from a rabid animal and timely administration of rabies immunoglobulin (RIG)/rabies monoclonal antibodies (RMAb) in all category III exposures to neutralize the virus at the wound site. ^11^

Therefore, thorough wound washing with prompt administration of cell culture vaccine and simultaneous administration of RIG/RMAbs in all category III exposures is almost invariably effective in preventing rabies, even after high-risk exposure. ^12^ It is also essential for animal bite victims to complete the full course of vaccination as recommended by the physician for full protection, as those who do not complete the full course of vaccines are still at risk of developing rabies. ^13^ However, compliance with anti-rabies vaccination among bite victims is still a major issue, as it spans over a period of 28 days. Therefore, the health behaviors of animal bite victims are important for the completion of PEP.

## Need for the study

To avail immediate and correct post exposure prophylaxis, animal bite victims should have the proper knowledge and attitude to seek proper health care services. Likewise, exposed individuals are supposed to complete the full course of anti-rabies vaccination for complete protection against the disease.

The present study was conducted to determine the health-seeking behavior of animal bite victims, along with their knowledge and attitude, the post exposure prophylaxis received by them, and their compliance with the full course of anti-rabies vaccination. The details regarding these important determinants are important to assemble new evidence for planning specific interventions to accomplish the task of eliminate rabies by 2030. ^14^

## Scope of the study

The study provided details regarding the health-seeking behavior of animal bite victims and assessed their knowledge and attitude toward rabies prophylaxis. It also provided details of the post exposure prophylaxis received by the patients and their compliance with the complete course of anti-rabies vaccination. This information is important for understanding the present situation and for the further implementation of specific strategies to eliminate rabies.

## Aim & Objectives

### Aim

To understand health-seeking behavior and post exposure prophylaxis received by animal bite victims.

### Objectives

1. To determine the health-seeking behavior of animal bite victims attending an anti-rabies clinic.
2. To assess the knowledge and attitude toward rabies prophylaxis and the perceived risk of disease transmission from different animals.
3. To describe the post exposure prophylaxis provided at the anti-rabies clinic and determine the compliance of study subjects to complete the course of vaccination.
4. To determine the clinical outcomes after receiving post exposure prophylaxis.

**Methods:** The study was conducted at the anti-rabies clinic, Preventive Medicine Unit, Kempegowda Institute of Medical Sciences (KIMS) Hospital and Research Centre, Bangalore, after obtaining Institutional Ethics Committee approval.

**Study subjects:** Animal bite victims who reported to the anti-rabies clinic, KIMS Hospital, and Research Centre for availing post exposure prophylaxis services and gave written informed consent to participate in the study.

Subjects were given information sheet in their understandable language to read and understand about the objectives of the study, methodology, possible side effects and benefits. Any queries raised by the study subjects were addressed by the investigator. Then, written informed consent from adults; informed consent from the guardian and informed assent was taken from adolescents and informed consent from the parents of children below 7 years who agreed to participate in the study were obtained (Annexure- I).

**Study period:** One and half years (January 2020 to June 2021).

**Study design:** A prospective longitudinal study.

**Sampling method:** Non probability type of purposive sampling technique.

**Sample size:** The sample size was calculated as per the compliance rate to complete course of anti-rabies vaccination, which is an important health behavior in post exposure prophylaxis; based on the published study as 77% ^15^; with 95% confidence level; allowable error (d) = 5% and assuming 15% dropouts.

$$\text{Sample size}:n=Z^{2}\;\alpha /2\;P\;\left(1-P\right)/d^{2}=3.84\;\times \;0.77\;\times \;0.23/0.039^{2}=447$$

where Z = Value from standard normal distribution table at α = 5% (95% confidence level).

$$P=\text{expected prevalence}\;\left(\text{Compliance to IDRV}=77\%\;\text{or}\;P=0.77\right).$$

1 – P = 0.23.

d = desired relative precision (5% of 77% = 0.039).

Assuming 15% of non-response rate 447 × 0.15 = 67

Sample size = 447 + 67 = 514 ≈ 525

As we included all patients who met the inclusion criteria during the study period, the net sample size was = 1058

### Inclusion criteria

➢ Animal bite victims who gave written informed consent.
➢ Study subjects who were available for follow-up.

### Exclusion criteria

➢ Animal bite victims who had already started post exposure prophylaxis elsewhere and seeking further management.
➢ Those with previous history of animal exposures and received post exposure prophylaxis or taken pre-exposure prophylaxis for preventing rabies.
➢ Bite victims < 18 years without accompanying parents/guardians.

**Figure 1.**
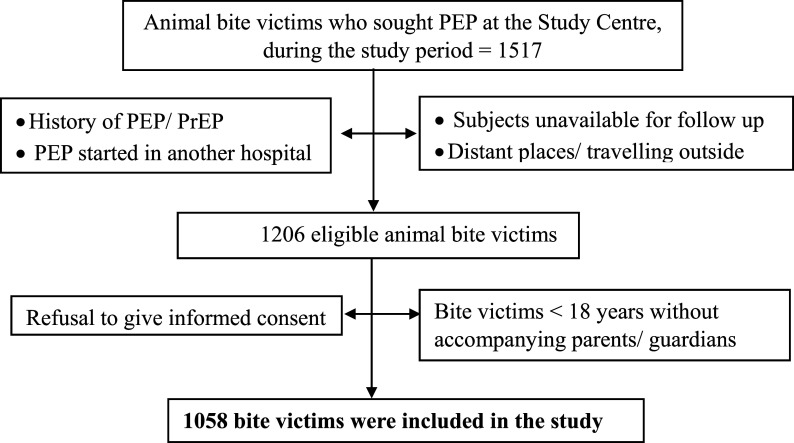
Flow chart showing the research participants recruited for the study.

### Institutional Ethics Committee clearance

The Institutional ethical Committee of KIMS was discussed ethical issues and approved the following.

• The study was conducted in compliance with the principles of the Declaration of Helsinki and following the ICH-GCP guidelines.
• Signed informed consent was taken from the study subjects ≥ 18 years of age.
• Written informed consent was obtained from parents or legal representatives for subjects of 2-17 years along with written informed assent from subjects aged 7-17 years.
• All subjects were monitored for ADRs, if any, and treated free of cost.
• Professional secrecy was maintained.

The Institutional Ethics Committee reviewed the proposed dissertation study entitled “A descriptive study on health seeking behaviour & post exposure prophylaxis received by animal bite victims at the anti-rabies clinic” by Dr Jithin Surendran, Post graduate student in the Department of Community Medicine under the Guidance of Dr Ravish HS, Professor of Community Medicine, Kempegowda Institute of Medical sciences, Bangalore, under Rajiv Gandhi University of Health Sciences, Karnataka during the meeting held on 27-10-2018. The members of the ethics committee were present at the meeting held on 27-10-2018 at 10:00 AM in Council Hall, Kempegowda Institute of Medical Sciences, Banashankari, Bangalore-560070. The approval reference number is KIMS/IEC/D106/2018, Dated 10-11-2018.

### Data collection

**Case Record Form:** All information pertaining to the subjects who had come for post exposure prophylaxis during the study period was recorded in a separate case record form. The case record form included information on age, sex, address, phone number, education, occupation, relevant past and present medical history, weight, physical examination, history of any allergy, intake of any medication, history of any animal bites, and characteristics of exposure.

Likewise, the details of health-seeking behavior and their knowledge and attitude toward rabies prophylaxis and perceived risk of disease transmission from different animals were also studied in detail.

### Clinical examination

A detailed clinical examination, including a general physical examination and systemic examination, was performed to determine the health status of the animal bite victim. Similarly, all wounds present in the study subjects were examined in detail.

### Categorization of bite wounds

The bite wounds were classified according to WHO categorization as shown below. ^11^

**Table:** 

Category	Type of exposure to a domestic or wild animal suspected or confirmed to be rabid or animal unavailable for testing	Recommended Post Exposure Prophylaxis
I	Touching or feeding animals, Licks on intact skin (No exposure)	None, if reliable case history is available ^a^
II	Nibbling of uncovered skin, Minor scratches/abrasions without bleeding (Exposure)	Administer vaccine immediately. Stop treatment if animal remains healthy throughout an observation period of 10 days ^b^/are proven to be negative for rabies by a reliable laboratory using appropriate diagnostic techniques. Treat as category III if bat involved.
III	Single or multiple transdermal ^c^ bites or scratches, contamination of mucous membrane/broken skin with saliva from animal licks; Exposures due to direct contact with bats (Severe Exposure)	Administer rabies vaccine immediately & rabies immunoglobulin, preferably as soon as possible. Rabies immunoglobulin can be injected up to 7 days after administration of first vaccine dose. Stop treatment if animal remains healthy throughout an observation period of 10 days or is proven to be negative for rabies by a reliable laboratory using appropriate diagnostic techniques.

^a^ If an apparently healthy dog or cat, in or from a low-risk area, is placed under observation, treatment may be delayed.

^b^ This observation period only applies to dogs and cats. Except for threatened or endangered species, other domestic and wild animals suspected of being rabid should be euthanized, and their tissues examined for the presence of rabies antigen by appropriate laboratory techniques.

^c^ Bites, especially on the head, neck, face, hands, and genitals, are category III exposures because of the rich innervation of these areas.

### Post exposure prophylaxis

Participants in the study received post-exposure prophylaxis (PEP) at the anti-rabies clinic in accordance with guidelines set forth by the NCDC, India. The above included a thorough cleansing of the wound with soap and running water for ten to fifteen minutes, a full course of intramuscular anti-rabies vaccination according to the Essen regimen, which requires one dose of vaccine on Days 0, 3, 7, 14, and 28, and the simultaneous administration of either rabies immunoglobulin, such as human rabies immunoglobulin (HRIG)/equine rabies immunoglobulin (ERIG) or rabies monoclonal antibody (RMAb), to all category III exposures according to the calculated volume in accordance with weight on day “0.”

### Mode of Administration of full dose of rabies immunoglobulin

Human rabies immunoglobulin (HRIG) was calculated at a dose of 20 IU/kg body weight, equine rabies immunoglobulin (ERIG) at a dose of 40 IU/kg body weight, and human rabies monoclonal antibody (RMAb) at a dosage of 3.33 IU/kg body weight.

All wounds were infiltrated with RIG/RMAb to neutralize the virus locally at the wound site, as the virus may be present in any of the wounds. As much of the calculated dose of RIG/RMAb, as is anatomically feasible, was infiltrated into and around all wounds. If any, the remaining was administered deep intramuscularly at a site away from the anti-rabies vaccination. If there were multiple wounds, then the volume required for infiltration of each wound was determined, and the total volume for infiltration was determined by appropriate dilution of the passive immunization, and all wounds were infiltrated.

### Assessment of safety to post exposure prophylaxis

All subjects were observed for half an hour, to rule out any possible immediate adverse drug reactions, both local and systemic. Then, all study subjects were given a follow-up card indicating the date of the next dose of vaccination and contact details of the anti-rabbit clinic and the study personnel to contact any issues. Adverse drug reactions, if any, were recorded during subsequent visits of the subjects for vaccination on days 3, 7, 14, and 28. All adverse drug reactions were treated free of cost at an anti-rabies clinic.

### Compliance to complete course of intramuscular anti-rabies vaccine

All study participants were informed about the subsequent dates of vaccination and telephonic reminders on the day of vaccination. However, if there were any dropouts in the natural course of vaccination.

All animal bite victims who had completed the recommended course of anti-rabies vaccination were deemed compliant; however, those who has stopped at any point during the recommended course (apart from those who stopped after three doses, in which case the dog or cat continued to be healthy and alive for at least ten days following the exposure) were considered non-compliant or dropouts. All non-compliant cases were recorded, and the reasons for incomplete vaccination courses were determined by interviewing the non-compliant bite victims or their guardians via telephone.

### Clinical outcomes after availing post exposure prophylaxis.

All study subjects were followed up for 6 months to determine their health status and clinical efficacy of post exposure prophylaxis. The survival of the exposed persons after the completion of rabies PEP and beyond the usual incubation period of the disease (i.e., six months, is an indicator of the clinical efficacy of the treatment.

### Data collection during COVID-19

The final stage of the study was conducted during the COVID-19 pandemic period. The subjects were advised to follow appropriate COVID behavior and comply with the local authority guidelines. The recruitment strictly followed the necessary precautions to prevent the transmission of the disease. The patients were followed-up until the end of the study to rule out the development of COVID-19 and its complications.

### Monitoring

During the study period, the study was monitored by a guide and an institutional ethics committee.

### Data analysis and statistical inferences

The data collected in the study were statistically analyzed using MS Excel and IBM SPSS statistics software package version 25.0. The results were computed using frequencies and percentages. Logistic regression analysis was performed to determine the association between the study variables and health-seeking behavior of the study subjects.

## Results

The study included 1058 animal bite cases, who visited the anti-rabies clinic, Preventive Medicine Unit, Kempegowda Institute of Medical Sciences, Bengaluru, to receive post exposure prophylaxis. The details of the study participants are as follows.

The age range of 18–59 years comprised the majority of study participants, making up 57.9% of), followed by children under the age of 18 with 33.1%). The majority of the study participants were male (60.1%), and 84.4% of the respondents reported being literate. According to the Modified B.G. Prasad scale, 46.6% of the study subjects fell under medium socioeconomic class, and 35.6% of the population were employed.

Dogs accounted for the majority (92%) of the biting animals, among whom 65% of them were stray animals. More than 80% of the biting animals either lacked vaccinations or were uncertain about their vaccination status. A significant number of exposures occurred after unprovoked encounters, and for 41.3% of the respondents, the outcome of the animal that bit them was undetermined.

One of the primary predictors of post exposure prophylaxis is health-seeking behavior after exposure. Fortunately, 93.3% washed their wounds right away, and three-quarters of them used detergent soap to scrub the exposed area. The application of virucidal agents or antiseptics is an important component of PEP, and 56% of the study participants used it. A total of 62.4% of study individuals attended another health care institution before coming to the anti-rabies clinic, while 30.8% arrived at the study setting directly after exposure. 74.3% landed at the study sites within one day of exposure, implying that a significant number received the PEP within 24 h ( Table 1).

**Table 1.** Health seeking behaviour of the study subjects (n = 1058).

Health seeking behaviour		Frequency	Percentage
Wound washed with	Water	281	26.6%
	Water & Soap	706	66.7%
	Not washed	71	06.7%
Time interval of wound wash	<1 hour	737	69.7%
	After 1 hour	250	23.6%
	Not done	71	6.7%
Antiseptics used	Yes	591	55.9%
	No	467	44.1%
Health care sought	Visited another health care facility	660	62.4%
	Visited AYUSH doctors	51	4.8%
	Consulted veterinarian	21	2.0%
	Came directly to the study Centre	326	30.8%
Duration of visit to ARC	<1 hour	77	07.3%
	>1 hour to <1 day	709	67.0%
	>1 day	272	25.7%

An essential component for receiving early and appropriate PEP is knowledge and attitude towards rabies prophylaxis. Subjects’ general knowledge of rabies was satisfactory, as evidenced by the largest percentage of precise responses (88.8%) for ‘animals that transmit the disease’ and the lowest percentage (41%) for ‘disease severity’. Despite a maximum of 41.4% for the total number of ARVs to be given in PEP and a minimum of 17% for PrEP, the study subjects’ comprehension of PEP fell short of expectations.

The attitude responses were satisfactory among study subjects which have a maximum right response of 92.1% for ‘availability of ARV’ followed by ‘rabies as a preventable disease’ (88.6%) and ‘importance of wound wash after exposure’ (82.6%). The attitude regarding pre-exposure prophylaxis was directly related to their knowledge regarding same as only 31% replied that PrEP was equally important in preventing rabies ( Table 2).

**Table 2.** Knowledge and attitude regarding prevention of rabies (n = 1058).

Rabies prevention		Frequency	Percentage
Knowledge	Heard about the disease	580	54.8%
	Severity of the disease	434	41%
	Animals causing rabies	940	88.8%
	Number of ARV doses for PEP	438	41.4%
	Site of vaccination	393	37.1%
	Role of rabies immunoglobulin	344	32.5%
	Number of ARV doses for PrEP	180	17.1%
Attitude	Severity of bite and disease transmission	717	67.8%
	Wound wash after exposure	874	82.6%
	Is it a preventable disease	937	88.6%
	Need for post exposure prophylaxis	726	68.7%
	Availability of ARV	974	92.1%
	Correct system of medicine for PEP	793	74.9%
	Need for pre-exposure prophylaxis	327	30.9%

The perceived risk of disease transmission by the study subjects was assessed based on a 1 to 5 Likert scale, in which 1 is the lowest rating and 5 is the highest rating. The perceived risk of disease transmission by various animals among the study participants varied from person to person. The perception was poor for risk of disease transmission through other animals, apart from dogs, viz. disease transmission by cats, livestock, mongoose, monkey, and other wild animals was <25%. Rodents that were not considered potential transmitters of rabies were rated by the respondents ( Table 3).

**Table 3.** Perceived risk of disease transmission by the study subjects (n = 1058) (1 = No/little risk of rabies to 5 = very high risk of rabies).

Biting animal	1	2	3	4	5
Dog	18 (1.7)	38 (03.6)	63 (06)	57 (05.4)	882 (83.3)
Cat	253 (23.9)	143 (13.6)	178 (16.8)	232 (21.9)	252 (23.8)
Livestock	289 (27.3)	315 (29.8)	217 (20.5)	120 (11.3)	117 (11.1)
Mongoose	183 (17.3)	167 (15.8)	368 (34.8)	217 (20.5)	123 (11.6)
Monkey	281 (26.6)	203 (19.2)	135 (12.8)	210 (19.8)	229 (21.6)
Bats	256 (24.2)	215 (20.3)	183 (17.3)	201 (19)	203 (19.2)
Rodents	272 (25.7)	207 (19.6)	285 (26.9)	165 (15.6)	129 (12.2)

Figures in parenthesis indicates percentage.

PEP was provided to those with Category II (ARV) or III exposures. In most of the cases, PVRV was the type of ARV administered, and more than 61% of the patients with Category III exposures decided to receive rabies monoclonal antibody as passive immunization, followed by ERIG (38%). Prior to the administration of ERIG, the subjects underwent a skin sensitivity test; however 89% of them showed no reactions. Based on the anatomical feasibility, RIG/RMAB was infiltrated into and around the wound site. In 90% of the study subjects, it was infiltrated locally, while the remaining study subjects received the required amount both locally and by an intramuscular systemic injection.

Compliance with the full course of the anti-rabies vaccine among the study participants showed a considerable drop off after each vaccination schedule. Out Of the 1058 subjects, 9.1% was the drop out for the 2 ^nd^ dose of vaccination, which came down to 11.7%, 18.8%, and subsequently to the lowest of 22.1% for the last and final dose of ARV.

A total of 234 dropouts and the reasons for dropout mentioned by the study subject after each telephonic interview were listed as long distance of travel, negligence, forgotten dates, interference with work timing, and school timings ( Table 4).

**Table 4.** Compliance to full course of anti-rabies vaccine among study subjects and reasons for drop out.

Compliance & Reasons to drop out		Frequency	Percentage
Compliance to Vaccination schedule (n = 1058)	1 ^st^ dose	1058	100%
	2 ^nd^ dose	962	90.9%
	3 ^rd^ dose	934	88.3%
	4 ^th^ dose	859	81.2%
	5 ^th^ dose	824	77.9%
Reason to drop out (n = 234)	Long distance of travel	55	23.5%
	Interferes with school timings	08	03.4%
	Negligence	58	24.8%
	Forgotten dates	56	23.9%
	Interfere with work timing	57	24.4%

The safety of post exposure prophylaxis was assessed in all study subjects up to day 28. The local adverse reactions included pain, erythema, itching, and swelling, and the systemic adverse events included body ache, fever, and malaise. All study subjects with adverse drug reactions were treated free of cost at the study center, and all subsided without any complications.

All study subjects were followed up for 6 months and were found to be healthy, showing that the post exposure prophylaxis provided at the anti-rabies clinic was effective in preventing the disease.

To assess the overall effect of the study variables on wound washing practiced as a health behavior, the multiple logistic regression model was applied to the variables that were statistically significant in the univariate logistic regression analysis. It was found that there was a significant association between the socioeconomic status of the animal bite victims, fate of biting animals, and type of wounds with their health behavior.

A multiple logistic regression model was applied to the study variables that were statistically significant in the univariate logistic regression analysis with regard to the time interval for receiving post exposure prophylaxis. A significant association was found between the socioeconomic status of bite victims, vaccination status, and fate of biting animals.

A multiple logistic regression model was applied to the study variables that were statistically significant in the univariate logistic regression analysis with regard to the association between the study variables and health care sought by bite victims. This showed that there was a significant association between the occupation of bite victims and vaccination status of the biting animals ( Table 5).

**Table 5.** Association between the study variables and health care sought by the bite victims (n = 1058).

Study variables		Adjusted Odds Ratio (95% CI)	SE	P-Value
Occupation	Employed	-	-	-
	Un employed	1.7 (1.1-2.7)	0.2	**0.02**
Vaccination status of animal	Vaccinated	-	-	-
	Un vaccinated	2.4 (1.3-4.5)	0.3	**0.004**

## Discussion

The present study provided details regarding the health-seeking behavior of animal bite victims and assessed their knowledge and attitude toward rabies prophylaxis and perceived risk of disease transmission. It also provided details of the post exposure prophylaxis received by the patients and their compliance with the complete course of the anti-rabies vaccine.

The health behavior in the present study showed that 93.3% of the subjects had washed their wounds, and 55.9% had applied local antiseptics. Similarly, a cross sectional study on epidemiology of animal bite cases attending the anti-rabies clinic of a rural tertiary care hospital of Haryana among 3897 animal bite victims revealed that only 54.1% animal exposed cases had washed their wound with either only water or with soap and water. ^16^ Another descriptive study on epidemiological determinants of animal bite cases attending the anti-rabies clinic in Sassoon hospital, Pune including 3226 animal bite cases showed that only 33.7% had washed their wounds with soap and water after the bite. ^17^

The present study also showed that 62.4% of the patients had visited other health care facility before coming to the anti-rabies clinic viz. corporation dispensaries, private hospitals, private clinics, etc.; 4.8% of them visited AYUSH doctors, and 2% consulted veterinarians. Likewise, 7.3% of them came to anti-rabies clinic for post exposure prophylaxis within an hour after exposure/bite and 67% of them came for treatment within 24 hours, whereas 25.7% came to anti-rabies clinic after 24 hours of exposure to seek post exposure prophylaxis. Similarly, a study from Kolkata of 257 people residing in rural areas showed that only 73.2% of the exposed individuals would like to go to allopathic doctors, whereas others would visit some local practitioners/religious practices. ^18^

The present study also showed that there was a significant association between some of the characteristics such as occupation of the bite victims (OR = 1.7; 95% CI 1.1-2.7); socioeconomic status of bite victims (OR = 3.9; 95% CI 1.5-10.3), vaccination status of the biting animal (OR = 2.8; 95% CI 1.7-4.6), and fate of the biting animal (OR = 1.5 95% CI 1.1-1.7) and the wound characteristics such as site of wound (OR = 16.5; 95% CI 10.8-23.9) and type of wound (OR = 1.6; 95% CI 1.2-2.3) with their health seeking behavior. All these studies showed that the health-seeking behavior of the exposed was inadequate and must be addressed through continuous social and behavior change communication (SBCC) activities. Increased awareness engages communities and empowers people to save themselves by seeking proper health care, thereby helping to eliminate dog-mediated human rabies by 2030.

The present study showed that the knowledge regarding prevention of rabies was incomplete, as only 54.8% had heard about the disease, 41% new about the severity of the disease, 41.4% knew about the number of anti-rabies vaccination doses to be taken after the bite, 37.1% knew about the site of vaccination, 32.5% knew about the need for rabies immunoglobulin for severe exposures, and 17% knew about pre-exposure prophylaxis. Similarly, the attitude of the study subjects was not satisfactory with regard to type of exposures transmitting disease (67.8%), need for first aid measures after exposure (82.6%), 100% preventable disease (88.6%), need for post exposure prophylaxis (68.7%), place of anti-rabies vaccine availability (92.1%), correct system of medicine for prevention of rabies (74.9%), and need and dosage for pre-exposure prophylaxis (30.9%).

Similarly, other studies also showed that KAP for the prevention of rabies was not adequate. In Mandya, Karnataka, a survey on medical school students’ awareness of rabies prevention indicated that only 45.1% of the respondents knew the extent to which rabies is throughout India and only 36.3% of them was aware how to safeguard themselves from dog bites. ^19^ A study of 220 schoolchildren found that their understanding of rabies transmission was only 33%, while their knowledge of animals transmitting the disease was 65%. ^20^ Similarly a study conducted on Accredited social health activists in rural Karnataka region had a poor understanding of animals spreading rabies (23.8%), the proper amount of ARV (25.7%), and the site of vaccine administration (54.1%). ^21^ These findings suggest that frequent health education sessions on rabies prevention at all levels are necessary to improve our understanding.

In the present study, a total of 97% of the study subjects received PVRV and remaining PCEC vaccine. Along with vaccine, 61% of the study participant preferred to receive RmAb and 38.2% of the study subjects received ERIG; among whom the skin sensitivity test was positive among 11% who developed mild hypersensitivity reactions. Patients with hypersensitivity were provided symptomatic treatment, and a full dose of RIG was administered.

Likewise, another study conducted at the antirabies clinic in Bangalore among 65 subjects who had wild animal exposures showed that all of them had received complete post exposure prophylaxis according to WHO, which included thorough wound wash (100%) and infiltration of rabies immunoglobulin; equine in 93.8% and humans in 6.2% cases. All study subjects received anti- rabies vaccination via intramuscular route using various types of vaccines i.e.: PCECV (60%), PVRV (33.9%), PDEV (4.6%), and HDCV (1.5%). ^22^

A full course of anti-rabies vaccine and RIG/RMAb is an important component of PEP, which has to be provided at all health care centers to prevent rabies, even after high-risk exposure. In the present study, the compliance with full course of antirabies vaccination was 77.9%. The compliance for vaccination doses slowly reduced from 90.9% for the 2nd dose on day 3 to 88.3% for 3rd dose on day 7, 81.2% to 4th dose on day 14, and 77.9% for the last dose on day 28. The reasons for the dropout were long distance of travel to the anti-rabies clinic, interference with school timings, negligence, forgotten dates, and interference with work timings. None of them stopped the vaccination course after day 10, even though the biting dog/cat was alive and healthy, as they wanted to complete the course.

Similarly, a multicentric study conducted across the country among 529 animal bite victims, showed that majority (54.4%) of the animal bite victims had Category III exposures; the overall compliance to complete course of vaccination was 78.8%, namely 65.9% for intramuscular rabies vaccination (IMRV) and 85.1% for IDRV. ^9^ Another cross-sectional study on compliance to anti-rabies vaccine among dog bite victims in an urban slum of Chennai showed that the compliance to complete course of vaccination was found to be 55.1%. The reasons for not completing the vaccination course were loss of wages, forgotten dates, and interference with school timing. ^23^

In the present study, among the 1058 study subjects who received post exposure prophylaxis, 103 (9.9%) had adverse drug events. The local adverse reactions included pain, erythema, itching, and swelling, and the systemic adverse events included body ache, fever, and malaise. All adverse drug reactions were mild and subsided spontaneously or with symptomatic medications. Comparably, a study carried out in Bangalore on 550 victims of animal bites who were attending an anti-rabies clinic showed that an overall 7.1% of ADEs were reported by the study participants. The most common local adverse events were pain (8.5%), erythema (7.3%), and itching (5.8%); the most common systemic adverse reactions were fever, malaise, headache, and body ache. ^24^ Likewise, a prospective, multicentric, post-marketing surveillance study on 168 subjects showed that immediate ADRs to ERIG administration were seen in 31.5%, including pain (30.4%), swelling (5.4%), pruritis (3.6%), induration (1.2%), itching, and erythema (0.6%). ^25^

Each of the studies showed that the adverse events to post-exposure prophylaxis (ADEs) were negligible and minor, and that they resolved on their own with symptomatic therapy without any further consequences. This indicates that PEP is safe for victims of animal bites. All subjects were followed up for six months and were found to be healthy, showing that the post exposure prophylaxis provided at the anti-rabies clinic was effective in preventing the disease.

## Conclusion

Rabies is a preventable disease with timely and appropriate post exposure prophylaxis, which includes thorough wound washing, full course of anti-rabies vaccination, and infiltration of rabies immunoglobulin/rabies monoclonal antibodies into all category III wounds and exposures.

In the present study, the health-seeking behavior of the exposed individuals, their knowledge and attitude toward the prevention of rabies, and the perceived risk of disease transmission by various animals was inadequate and must be addressed appropriately.

Adverse drug reactions for PEP were seen in a minimal number of study subjects, which were mild and subsided on their own or by symptomatic treatment, which showed that post exposure prophylaxis for all animal bite victims is safe and tolerable. Likewise, all study subjects were healthy and alive after a follow-up of six months, showing that the post exposure prophylaxis provided was effective in preventing the disease.

## Recommendations

1. Knowledge and health-seeking behavior must be improved through continuous social and behavior change communication (SBCC) activities. This engages communities and empowers people to save themselves by seeking proper healthcare services.
2. The compliance to full course of anti-rabies vaccination has to be assured in all animal bite victims, by proper health education, thereby motivating them and providing the dates of next vaccination in the treatment card/telephonic remainders.
3. Post exposure prophylaxis services for all animal exposures must be provided free of cost by the government at all health centers throughout the year under the universal health coverage program.

## Limitations of the study

1. Rabies virus neutralizing antibody (RVNA) analysis to assess the immunogenicity of the vaccine was not performed because of cost feasibility.
2. The biting animals could not be followed up to confirm their health status owing to logistic reasons.

## Data Availability

Figshare: THESIS EXCEL DATA ENTRY.xlsx, https://doi.org/10.6084/m9.figshare.24709566.v1. ^26^ Figshare: Extended data, https://doi.org/10.6084/m9.figshare.25151543.v1. ^27^ Data are available under the terms of the Creative Commons Attribution 4.0 International license (CC-BY 4.0).
