# Supplementary material for: A prospective study on health seeking behaviour and post exposure prophylaxis received by animal bite victims at the anti-rabies clinic in a tertiary care centre of urban Bangalore
Source: F1000Res. 2024 Mar 11;13:175. [Version 1] doi: 10.12688/f1000research.145673.1 (PMC11249507; doi:10.12688/f1000research.145673.1)
Supplement: Peer review history

# Reviewer report: Reviewer response for version 1

**DOI:** 10.5256/f1000research.159650.r256099

**Published:** 2024-07-15

**Authors:** Dr Harish B R

**Affiliations:** 1 Mandya Institute of Medical Sciences, Karnataka, India

Brief Description and relevance: This study focuses on the knowledge and attitude of animal bite victims in seeking post exposure prophylaxis and also on their compliance to the full course of post exposure prophylaxis. The study reveals compliance to be around 77.9% with various reasons for drop out being distance, negligence, forgetfulness and other commitments were and knowledge of rabies and its prevention among animal bites to be low at 54.8%. The study notes the need for sustained social and behavioral change communication (SBCC) to improve the health seeking behavior in animal bite victims and their knowledge of rabies prevention.

- Grammatical errors need to be rectified, otherwise, it is a pertinent study. It tries to answer the gap in knowledge and attitude of animal bite victims in seeking timely PEP.
- Aim and objectives are in line with the title and the study.
- Sampling method and sample size calculation is as required.

Is the work clearly and accurately presented and does it cite the current literature?

Yes

If applicable, is the statistical analysis and its interpretation appropriate?

Yes

Are all the source data underlying the results available to ensure full reproducibility?

Yes

Is the study design appropriate and is the work technically sound?

Yes

Are the conclusions drawn adequately supported by the results?

Yes

Are sufficient details of methods and analysis provided to allow replication by others?

Yes

Reviewer Expertise:

Prevention of Rabies

I confirm that I have read this submission and believe that I have an appropriate level of expertise to confirm that it is of an acceptable scientific standard.

## References

[1] : A prospective study on health seeking behaviour and post exposure prophylaxis received by animal bite victims at the anti-rabies clinic in a tertiary care centre of urban Bangalore. *F1000Research* .2024;13: 10.12688/f1000research.145673.1 10.12688/f1000research.145673.1 PMC1124950739015143

# Reviewer report: Reviewer response for version 1

**DOI:** 10.5256/f1000research.159650.r267093

**Published:** 2024-05-24

**Authors:** Dilceu Silveira Tolentino Júnior

**Affiliations:** 1 René Rachou Institute, Minas Gerais, Brazil

This is a very interesting study to seek to understand the health-seeking behavior of victims of animal bites, their knowledge and attitude towards anti-rabies prophylaxis, as well as evaluating the level of adherence to the complete cycle of anti-rabies vaccination. The study design is appropriate and the work is technically sound for the proposed objective. Statistical analysis and its interpretation are appropriate for the results obtained. I believe that all source data underlying the results is available to ensure full reproducibility and can support future studies. The conclusions are adequately supported and well represented by the results section. The work is well presented in a clear and precise way, but I suggest that the authors include some more current bibliographic references (from the last 5 years) to better contemporize the present work.

Is the work clearly and accurately presented and does it cite the current literature?

Yes

If applicable, is the statistical analysis and its interpretation appropriate?

Yes

Are all the source data underlying the results available to ensure full reproducibility?

Yes

Is the study design appropriate and is the work technically sound?

Yes

Are the conclusions drawn adequately supported by the results?

Yes

Are sufficient details of methods and analysis provided to allow replication by others?

Yes

Reviewer Expertise:

Researcher in the field of human and animal rabies (neglected diseases).

I confirm that I have read this submission and believe that I have an appropriate level of expertise to confirm that it is of an acceptable scientific standard.

# Response

**Published:** 2024-05-25

**Authors:** DR JITHIN SURENDRAN

**Affiliations:** COMMUNITY MEDICINE, KMC Mangalore, MANGALORE, KARNATAKA, India

Thank you very much for your thorough review and valuable feedback. I appreciate your positive evaluation of my study on health-seeking behaviour of animal bite victims and their knowledge and adherence to anti-rabies prophylaxis.

 I acknowledge your suggestion to include more recent bibliographic references from the last five years. I agree that incorporating these references will enhance the contemporary relevance of our work. 

 Thank you once again for your constructive comments and insights.
